# The Influence of Hospital Policies on Clinicians’ Decisions to Withhold or Withdraw Life-Sustaining Treatment

**DOI:** 10.1016/j.chest.2025.06.036

**Published:** 2025-07-03

**Authors:** Gina M. Piscitello, Edlyn Lopez Wolwowicz, Michael T. Huber, Kelly C. Vranas, Donald R. Sullivan, Katrina E. Hauschildt, Patrick G. Lyons

**Affiliations:** a Division of General Internal Medicine, Section of Palliative Care and Medical Ethics, Pittsburgh, PA; b Palliative Research Center, University of Pittsburgh, Pittsburgh, PA; c Division of Pulmonary, Allergy and Critical Care, Oregon Health & Science University, Portland, OR; d Division of Geriatrics and Palliative Medicine, Miami, FL; e Institute of Bioethics and Health Policy, University of Miami, Miami, FL; f Health Services Research & Development Center to Improve Veteran Involvement in Care (CIVIC), VA Portland Health Care System, Portland, OR; g Division of Pulmonary and Critical Care Medicine, Johns Hopkins University School of Medicine, Baltimore, MD

**Keywords:** clinician behavior, hospital policy, life-sustaining treatment

## Abstract

**Background:**

There is considerable variation in clinicians’ approaches to decisions to withhold or withdraw life-sustaining treatment (LST) across US hospitals. These differences are not explained by patient preferences alone and are likely influenced by other factors (eg, hospital policies, hospital culture, state laws, medical society guidelines).

**Research Question:**

How do hospital policies influence clinician approaches to decisions to withhold or withdraw LST among patients admitted to an ICU?

**Study Design and Methods:**

We conducted semistructured interviews with ICU nurses and physicians at 3 geographically diverse hospital systems across the United States between July and October 2024. We asked clinicians about their experiences with, and perceptions of, hospital policies on withholding or withdrawing LST and the relationship between these policies and clinician decision-making in ethically challenging scenarios.

**Results:**

We interviewed 10 nurses and 8 attending physicians with a median of 5 years (range, 2-36 years) in practice. Clinicians described limited awareness of, and familiarity with, their hospital’s policies that addressed withholding or withdrawing LST. Clinicians with knowledge of these policies could identify their location but described barriers to accessing them. Although clinicians perceived hospital policies as helpful in some ways (eg, legal protection, ethical guidance), they viewed them as neither acknowledging nor addressing sociodemographic disparities or clinician value judgments in LST decision-making. Perceptions varied about whether clinicians followed their own hospital policy guidance when making decisions to withhold or withdraw LST.

**Interpretation:**

Our results show that clinicians lack detailed understanding about their hospitals’ policies that address withholding or withdrawing LST and perceive these policies as having limited applicability to clinical practice. These findings suggest that hospital policies may have little influence on clinician behavior in addressing decisions to withhold or withdraw LST in ethically challenging scenarios.

**Take-Home Points**

**Study Question:** How do hospital policies influence clinician approaches to decisions to withhold or withdraw life-sustaining treatment (LST) among patients admitted to an ICU?

**Results:** Our qualitative study of ICU nurses and physicians across 3 geographically diverse US hospital systems found clinicians lack detailed understanding about their hospitals’ policies that address withholding or withdrawing LST and perceive these policies as having limited applicability to clinical practice.

**Interpretation:** These findings suggest that hospital policies may have little influence on clinician behavior in addressing decisions to withhold or withdraw LST in ethically challenging scenarios.

Clinician approaches to decisions to withhold or withdraw life-sustaining treatment (LST) in the ICU vary across US hospitals.^1^ This variation has the potential to cause differences in patient outcomes across hospitals and contribute to sociodemographic disparities in the provision of LST.^2^^,^^3^ Differences in clinician approaches cannot be explained by patient preferences alone, with other factors such as hospital policies, hospital culture, state laws, and medical society guidelines likely contributing.^1^^,^^4^, ^5^, ^6^

US hospital policies for addressing decisions to withhold or withdraw LST also vary widely across hospitals and may influence variation in clinician practices across hospitals.^7^ Previous studies have found inconsistent alignment between clinician actions and detailed hospital policy guidance for non-LST decisions.^8^^,^^9^ Prior work has also examined clinician attitudes toward do-not-resuscitate treatment decisions and their alignment with broad ethical principles outlined in hospital policies.^10^ However, this work did not assess LST decision-making beyond CPR. It also did not assess how clinician behavior in LST decision-making may be influenced by discrete actions recommended within hospital policies (eg, providing the patient with a second medical opinion if they disagree with the clinician’s decision), nor examine how hospital policies may influence sociodemographic disparities in clinician decision-making.

Understanding how clinician behavior may be influenced by hospital policies would illuminate whether hospital policies may be leveraged to improve consistency in clinician behavior and reduce sociodemographic disparities in end-of-life care. This qualitative study thus aimed to understand how hospital policies may influence clinician approaches to decisions to withhold or withdraw LST in the ICU.

## Study Design and Methods

### Study Design and Setting

This multisite qualitative study explored critical care nurses’ and physicians’ perspectives about hospital policies that address decisions to withhold or withdraw LST. We interviewed participants at 3 geographically diverse academic hospital systems in the United States. These systems included private and public hospitals that care for racially/ethnically diverse, socioeconomically diverse, and rural patients, and were located in the Northeast, South, and West regions of the United States. Each hospital system had established institutional policies addressing decisions to withhold or withdraw LST that were available to all clinicians. This research study was determined to be exempt by the University of Pittsburgh institutional review board (STUDY23100042) and the Oregon Health Sciences University Institutional Review Board (STUDY00027237). We used the Consolidated Criteria for Reporting Qualitative Research (e-Table 1) to guide our study and description of results.^11^

### Participants

We leveraged purposeful sampling to recruit ICU nurses and physicians by email who varied by experience and ICU type. Physicians were eligible if they spent ≥ 5 wk/y as the attending physician of record for patients in the ICU. Nurses were eligible if they worked full-time in the ICU. Consultant physicians and physicians in training were not eligible because we chose to focus on those with direct responsibility for decisions to withhold or withdraw LST.

### Semistructured Interviews

Our research team included palliative care and critical care physicians, a medical sociologist, and an experienced qualitative research associate. Three members of the research team created the interview guide based on recent literature and direct experience in the clinical environment (e-Appendix 1).

The interview guide included questions to identify participant knowledge about hospital policy content, location, and perceptions about how these policies are helpful or limited. It explored how clinicians approach decisions to withhold or withdraw LST in ordinary clinical practice and in 3 ethically challenging patient care scenarios. These scenarios included the following: (1) a case of potentially inappropriate treatment where there is conflict between the patient’s surrogate who requests LST and the physician who declines to provide the LST, (2) a case of physiological futility where there is conflict between the patient’s surrogate who requests LST and the physician who declines to provide the LST, and (3) a case of an unrepresented patient, with no surrogate who can speak their behalf, who is receiving LST and has a severe brain injury. It also assessed whether hospital policies may influence sociodemographic disparities in decisions to withhold or withdraw LST. We piloted the interview guide with 3 clinicians and iteratively revised it as we completed interviews to improve question clarity and assess insights that emerged from earlier interviews.

### Data Collection

We used teleconferencing software (Cisco Webex; Cisco Systems, Inc) to conduct interviews approximately 30 to 60 minutes in length and recorded audio from these meetings. All interviews were conducted by an author (E. L. W.) with significant experience conducting health services research interviews who had no prior relationship with participants. We asked participants to self-identify their demographics (gender and race/ethnicity) and report their clinical role (ie, nurse or physician), number of years in clinical practice, type of ICU in which they primarily work (eg, cardiac, medical), and their hospital characteristics (eg, location, public/private).

### Data Analysis

We transcribed interview recordings verbatim using a combination videoconferencing transcription services using Cisco Webex, professional transcriptionists, and manual transcription, and then deidentified these transcripts. We used qualitative software for coding and analysis (NVivo Version 14; Lumivero). We used a grounded theory methodology and leveraged mixed inductive and deductive approaches for code development. We used deductive methods to create an initial code book, based on known components of hospital policy content. We expanded this codebook as we gained new insights from our data. Two research members (E. L. W. and G. M. P.) trained in qualitative analysis independently coded 2 interview transcripts using this initial codebook and met with a sociologist (K. E. H.) to compare, refine code definitions, discuss emerging inductive codes, and reach consensus on the codebook and code application. They then coded the remaining transcripts, double-coding 25%, and met multiple times to resolve discrepancies and add new codes through discussion to consensus. We identified themes from our codebook and discussed with the research team. We recruited participants until reaching thematic saturation. Once themes were identified, we compared participant responses to how clinicians approach decisions to withhold or withdraw LST in 3 ethically challenging scenarios with the expected actions for these scenarios detailed in participants’ hospital policies, state laws, and in medical society consensus policy statements.^12^^,^^13^

## Results

Of the 18 interviewed clinician participants, 10 (56%) were nurses and 8 (44%) were physicians. Two additional individuals who agreed to participate (1 nurse and 1 physician) did not attend scheduled interviews. Participants were from hospital system A (33%), B (44%), and C (22%) respectively. Participants’ median time in practice was 5 years (range, 2-36). Participants worked in medical (n = 11, 61%), neuro/trauma (n = 4, 22%), cardiac (n = 2, 11%), or cardiothoracic (n = 1, 6%) ICUs (Table 1). Interviews lasted a median of 43 minutes (interquartile range, 39-55).

**Table 1:** Participant Characteristics (N = 18)

Characteristic	Value
Gender	
Women	16 (89)
Men	2 (11)
Race/ethnicity	
Caucasian/White	17 (94)
Hispanic	1 (6)
Clinician type	
Nurse	10 (56)
Physician	8 (44)
Years in clinical practice	5 (3-7)
Type of ICU	
Cardiac	2 (11)
Cardiothoracic	1 (6)
Medical	11 (61)
Neuro/trauma	4 (22)
Hospital system	
A	6 (33)
B	8 (44)
C	4 (22)
Type of hospital	
Academic with residents and/or fellows present	18 (100)
Private	8 (44)
Public	10 (56)

Data are presented as No. (%) or median (interquartile range).

We identified 5 themes from the interviews: (1) clinician perspectives about hospital policies which address withholding or withdrawing LST, (2) limitations in clinician knowledge about these policies, (3) clinician perceptions about how these policies are used in practice, (4) perceived sociodemographic disparities in decisions to withhold or withdraw LST, and (5) varying alignment of clinician-reported approaches to decisions to withhold or withdraw LST with hospital policies, state laws, and medical society consensus guidelines.

### Clinician Perceptions About Hospital Policies

When clinicians were asked about how their hospital policies may influence clinician practice, some clinicians perceived their hospital policies as helpful because they prevent overtreatment of patients, legally protect clinicians, and provide guidance to do what is best for the patient. One nurse stated, “I think they give us guidelines and like safety rails so that we do everything as correct as possible to do what’s best for the patient but then also keep staff safe from a legal and moral perspective.” A physician stated, “When there’s a disagreement, the policy is helpful in that it helps us make sure, feel comfortable in, and probably prevents people from overtreating because of fear of litigation.”

In contrast, some nurses thought that their hospital policies were lacking because they were out of date, lacked important nuance, or were inconsistent with other hospital policies. For instance, 1 nurse stated, “A lot of the policies contradict each other and also a lot of them are out of date.”

### Limitations in Clinician Knowledge About Hospital Policies

All clinicians described limitations in their knowledge about hospital policies that address withholding or withdrawing LST.

Some clinicians were unaware of the existence of these policies. One physician stated, “If they exist, this is news to me and I’ve been here for 10 years.”

Others knew about their existence, but lacked detailed understanding about the content of these policies. A physician stated, “I don't know if anybody has actually fully read, you know, it's whatever or how many pages it has, but understands the breadth of it.” A nurse added, “I don’t know necessarily what the policies in specifics say about withdrawing LST.”

Limitations in detailed understanding were often associated with difficulty finding these policies on internally facing hospital system websites because of reasons such as not knowing which search terms to use to find the policy.

A physician stated, “Oh, it’s I mean it’s doable to, to find them but you know [it] probably requires a little bit of like crafting of different types of language to figure out exactly like what something like that is called.” A nurse added, “I do know how to get to the webpage to look for them, but it is pretty difficult to search … we’ve had these like issues where for 6 hours nobody can like figure out what the proper one is and one kind of says one thing and one kind of says another.”

### Clinician Perceptions About How Hospital Policies Are Used in Practice

Although participants identified clinician decisions to withhold or withdraw LST are influenced by factors such as futility of medical treatments, patient quality of life, and patient and family preferences, no participant discussed hospital policies influencing these decisions until being directly prompted to share their perspectives about their policies.

Once prompted, clinicians varied in their perspectives about how these hospital policies are used. Some participants viewed hospital policies as followed consistently in clinical decision-making. According to a physician, “I would say [these policies are followed] fairly consistently. I think that we may interpret it slightly differently, but we consistently use the hospital policy … to determine the beneficial or non-beneficial nature of care.” A nurse mentioned, “I think that we do a good job [following hospital policy] but like from the care team perspective, mostly because since I’m a leader on the unit, we’re not reviewing any cases where we may have done something wrong in withdrawing or withholding care.”

In contrast, other participants thought that policy adherence was limited or dependent on the physician team specialty. According to a nurse, “Maybe the providers are using policies and just not telling us about it, but I feel like I’ve never heard a provider reference a policy.” A physician added, “I think that’s very specialty dependent. I believe all my colleagues from the same specialty are very good about it because it’s our daily bread or it’s a very common topic.”

Finally, others expressed uncertainty as to whether policies were followed or not. One nurse stated, “I hope we do it correctly all the time … we must use them as they are to be used because I’ve never gotten in trouble for the care that we have done or not done. You know what I mean?”

### Perceived Sociodemographic Disparities in Decisions to Withhold or Withdraw LST

Most clinicians shared concerns that sociodemographic disparities in decisions to withhold or withdraw LST exist in clinical practice, with specific concern about these disparities by patient age, race, and language. Moreover, many clinicians observed barriers in addressing these disparities (Table 2).

**Table 2:** Clinician Perspectives About Sociodemographic Disparities in Clinician Decisions to Withhold or Withdraw LST

Themes	Representative Quotation
Concern for sociodemographic disparities in clinician decisions to withhold or withdraw LST	“I think we are overtly favoring the young. … I think, you know, a single man’s life is probably just as valuable to him as a, as a younger mother, but, we don’t talk about it that way or at least not everybody does. … There are occasionally times when I’m in meetings where people will get upset and emotional and say, this is a young man we have to try or this is a young woman we have to try.” -Physician “I’ve had very similar patients who’ve had bad infective endocarditis dying from their infective endocarditis who need valve replacement in order to save their life, and I feel like I’ve seen scenarios where we’ve gotten a White patient and a Black patient who look very similar, like who are systemically both very sick, and for whatever reason, we are offering the White patient lifesaving surgery and we really don’t even offer the Black patient surgery.” -Physician “I feel when there is a language barrier, I feel that inherently makes things more challenging, especially if there are no family that are easily found, or the family may only speak a certain language, and maybe we just haven’t actually been able to communicate with them. Maybe we’ve left messages, but they don’t understand them, things like that. I feel there are far more instances of bias in that setting.” -Nurse “I think we worry about the disparities in patients who have a lot of family or other people to advocate for them versus those that do not as well in the relationships and how those people connect with the ICU team.” -Physician “Last week my attending was talking about that [disparities in decisions to withhold and withdraw LST] because there was an article that came out that said that physicians withdraw care earlier on patients without insurance, which like as a group we were kind of like wow, because my physicians don’t even know if my patients have insurance, like it’s never really anything that we discussed. … Isn’t for not because somebody says that she’s not worth it because she doesn’t have insurance. Like we don’t, I don’t, I, that has never been a part of our conversation. My physicians don’t even want to know if they have insurance. I mean I live in the trauma world. So a lot of my patients don’t have insurance, like, I. It just boggles me I, I, I, I don’t, it just boggles my brain.” -Nurse “… [A]s a clinician, you’re always thinking about like how is this person going to end up after we’re done with that? So I think, you know, we kind of are biased in making decisions like is this going to be a good quality of life for this person? Like, are we doing all of this so they can like lay in a bed somewhere forever? And I think that that does create some conflict at times between care teams and families because we feel like families a lot of times will come up and be like, will they be fine as long as they can like, you know, open their eyes and see their grandchildren. Whereas we as care providers are like, is that really a suitable quality of life that we’ve gotten you through all of this and then you just go in a nursing home and like lay there for the rest of your life.” -Nurse “I think that we really have to check our own bias, and that is something that is difficult and requires intention, and we have had some practitioners who make assumptions about what other people would accept as their good quality of life or acceptable quality of life and I do think that it’s important for us to remember that, not everybody thinks the same way about this. It’s tough. For the most part within the ICU teams, we work well enough together and we have open lines of communication that we can check each other for our bias and for our assumptions. I know that there have been cases in the past where one of my colleagues has called palliative care [and] palliative care has come in, made some general assumptions about, quality of life … that the ICU team did not agree with. Specifically with a patient with traumatic brain injury … it leads to a lot of difficulty for the family because the ICU team is saying, I know they look really bad now, but the recovery from traumatic brain injury is months to years long and you need to give him a chance. And palliative care will be like, ‘oh, he looks terrible, he's suffering, we should stop.’ And that, that I think has been very problematic and I think it’s related to a lack of education about the underlying problem … so we’ve really tried hard to educate the palliative care teams and the palliative care fellows and the … hospitalist teams to try to understand, you know, neuroprognostication.” -Physician
Have not observed sociodemographic disparities in clinician decisions to withhold or withdraw LST	“I don’t necessarily notice any disparities when it comes to socioeconomic status, I guess in my practice. I feel like we treat everyone the same no matter what that status is. I just don’t feel like I have a lot of experience in seeing like the disparities.” -Nurse “I would say … I haven’t seen it, I haven’t seen any disparities in this aspect.” -Nurse “I personally would say I have not experienced [sociodemographic disparities] … I feel like we are very aggressive in all populations regardless of, you know, income [or] racial differences, I feel like any of that, I feel like we are very, very aggressive. I feel like maybe more aggressive than we should be in some cases. So it has not been my experience that we’ve withheld treatments, on different populations. I feel like we even offer it at times too much more care than we should to any in all the populations.” -Nurse
Barriers to addressing sociodemographic disparities in decisions to withhold or withdraw LST	“As a sort of us [as] a whole looking at populations to see how we’re doing, I'm not sure that the hospital is doing something like that … looking to see is like the same number of people getting transplants from like inappropriate proportions [of] the community.” -Physician “I’m not aware of any specific approaches the hospital takes to try to reduce those disparities or make people aware of those disparities … I’m not aware that they’re routinely evaluated.” -Nurse “I don’t know if there’s data I don’t know if we collect data. I don’t get the sense that we have great interventions to minimize these disparities.” -Physician “When I was at our smaller community hospital, I felt like there was a lot more, there was a lot more, decision-making … where race really played a role or insurance data has played a role. I see it a little less so at the big academic hospital to tell you the truth, but I think it does happen. And I don’t think our hospital does a great job of trying to minimize that.” -Physician “So the demographic data is collected, but it’s not, I don’t believe that it’s utilized to analyze disparities or anything.” -Nurse “So I think where the barrier lies is that I just, I don’t think that we’re aware, that we’re consciously aware that we are creating these barriers … I don’t know one’s going into being like, I’m gonna communicate differently because their race is X, you know what I mean? Like it’s this unconscious thing that happens … so, I think the biggest barrier is bringing this to light and then try making clinicians aware that their decision-making act is actually based on that.” -Physician

LST = life-sustaining treatment.

No clinicians thought that their hospital policies addressed these disparities. According to a physician, “I don’t think that they [address the use of clinician value judgments in medical decision-making]. I think they would if we were doing it badly, but as long as you’re practicing within a realm of like pretty normal and no one’s complaining then I don’t think that they do.” A nurse added, “I wouldn’t say that [the policies address disparities in decisions to withhold or withdraw LST] … I wouldn’t say that we pull out policies whenever there’s disparities like that.”

### Varying Alignment of Clinician-Reported Approaches to Decisions to Withhold or Withdraw LST With Hospital Policies, State Laws, and Medical Society Consensus Recommendations

We asked participants to describe how clinicians at their hospital respond to 3 ethically challenging patient cases involving decisions to withhold or withdraw life-sustaining treatment (Table 3). Although the perceived approaches to these scenarios were similar across hospitals, there were some notable differences. For example, although participants at all 3 hospital systems stated that some clinicians would not honor a surrogate’s request to provide LST in cases of physiological futility, only participants at 1 hospital system reported that agreement by 2 physicians was needed in this scenario.

**Table 3:** Clinician Perspectives About How Clinicians Approach Ethically Challenging Scenarios Within Their Hospital

Themes	Representative Quotation
Approaching cases of physiological futility	
Communicate with family	“Explaining that we would, you know how the whole process is, we will break ribs, the outcome will be the patient will not come out of the cardiac arrest. And if it does most likely it will be kept on the ventilator for X amount of time and it won’t recover, we, the way we approach it is that, you know, we, like I said, we always transparent with them and we ask them, do you want us to do chest compression? Yes or no?” -Nurse “We just kept talking to the wife … the patient … had actually had already arrested, but the wife wanted to continue because he’s always gotten better. So I had the postcardiac arrest team and I had my critical care medicine attending, and we just kept describing what had happened to the wife. And that everything we were doing was not working and that he was gonna die, and that there was nothing more for us to do, and she had said we she wanted us to do CPR and then they told them that that would be unsuccessful ...and probably cause more damage due to like lack of oxygen and brain function and stuff like that. And she kept repeating that she needed him to be alive.” -Nurse
Communicate reason why CPR should not or will not be offered to family	“Discuss in an empathetic and caring manner why we’re not going to do CPR, because there is no way for that to extend their life, it would only extend their suffering and death.” -Physician “Be really blunt and clear that this was not going to prolong their life and that they were dying regardless of what we did.” -Physician “First trying to have the, this, you know, discussion again with the surrogate explaining, you know, that they’re dying because of a respiratory cause, and that the CPR does not give us any additional ability to support their respiratory function beyond what they’re receiving and so won’t work.” -Physician “I’d say people just don’t want to make the choice themselves and so when you tell them you’re not offering it [CPR], they are relieved by that because now it’s my fault, and I’m absolutely willing to bear that burden for someone so that they don’t have to live with wondering if they made a bad choice, and so I actually have had very few arguments about not offering CPR with people once it’s explained, carefully and, you know, with empathy and, and that being said, you have to time for those conversations.” -Physician
Two physician documented agreement with unilateral DNR decision in the electronic medical record	“It seems pretty straightforward that unfortunately this would be a futile treatment and therefore should not be offered. And in this case especially in [state redacted], we do have two physicians who need to sign a, an order like this and we can’t actually put the order in the computer. We can put and document two terminal notes and then our hospital staff is still required to initiate CPR. Until a physician comes and requests that because of this being a terminal condition and that this is causing more harm than good that therefore we should stop.” -Physician “Because of some quirks in legislation, we would not be putting a DNR order in, into the medical record. What we would be doing is we would have multiple physicians document that this kind of care would be inappropriate, it would be non-beneficial. Again, we would refer to the hospital policies about non beneficial care … if in our judgment this was a futile scenario or scenario when the association would be non-beneficial, we can at the onset of this when it actually starts happening, we can instruct the team at that site not to do any resuscitation.” -Physician
Do not perform CPR	“Right, so, we never have to provide medical treatments that we do not think are beneficial for the, for the patient. However, we are not allowed to unilaterally place a DNR in the chart. If, if the surrogate does not agree with the DNR order, we are not, we cannot put that on the chart. However, the instructions to the medical team and to, you know, our fellows and residents and for the attendings is that if the patient goes into cardiac arrest, the clinical team can decide that it’s non-beneficial and not provide CPR. So basically we walk in the room and stop any code that may happen because it’s not beneficial to the patient.” -Physician “There’s been very limited time where we think it’s futile. The family thinks that it is important and that we still don’t offer it, which I think causes a great amount of moral distress, especially for us at the bedside.” -Nurse “I would not offer CPR in this case, and then I would continue to discuss in an empathetic and caring manner why we’re not going to do CPR, because there is no way for that to extend their life, it would only extend their suffering and death, and I would say about half of my colleagues would do the same and the other half would be likely to just provide a short, very abbreviated course of CPR.” -Physician
Perform CPR	“At the hospital I found that we will still keep them full code according to their family’s requests. I’ve never experienced a provider putting in a DNR DNI order against a family member;s will or, you know, surrogate's will.” -Nurse “So in the ICU is common practice to bring to all the family and ask them where their wishes are if they want to be the resuscitated or not … we cannot go against their wishes and we cannot place a DNR order without the family say.” -Nurse “I think the typical practice would probably be that we would keep them full code per the surrogate’s request.” -Physician
Perform limited trial of CPR	“I guess I see more commonly that we will do CPR despite thinking that it is futile. And we will say we’re going to do a time-limited one or three rounds or whatever.” -Nurse “As the bedside nurse, I’m often the first one that’s on the scene for that. And so we will kind of say like, ‘Hey, when this happens, we’re just going to do a short … generally at least one pulse and rhythm check within the ACLS kind of pathway.’” -Nurse “And, and we have several times we’ve spoken with the family and despite all of that, the patient gets being full code, but we do everything, we respect all the decisions and, you know, we try to do what’s best for the patient, but sometimes, you know, the family does not understand in that case, but we still respect that and we performed the CPR but not for a long time.” -Nurse “I would rather do something that I think is futile but short than, than make them bear the guilts of having failed to advocate, right? Right.” -Physician
Have family watch code	“And I think if they still were absolutely insistent that we performed CPR, I would probably honor that wish but very quickly bringing them into the room so they could see what was happening. And hopefully they would be willing to have us stop rather quickly.” -Physician “Ideally, they’re there and they can see that we’re trying and hopefully withdraw care or we’ll give it those 10-15 minutes, ask if the room, if there’s anything else we can do and then call time of death.” -Nurse “What we did was just have her come into the room with us while we were working on him and showing her everything that we were doing and explaining what we were doing and that it wasn’t working and there was nothing more for us to do.” -Nurse
Approaching cases of potentially inappropriate treatment	
Communicate with family	“We will continue kind of having care conferences where to kind of really push the family not to do this.” -Nurse “Communicating both with the family members to understand more of the patient’s wishes and the decision or the information that led them to make that decision on behalf of the patient. And in this type of scenario, the physician may offer it anyways with the documented and very thorough going through of all the risks and benefits and having the family member repeat back understanding of the risks and benefits” -Nurse “In in this situation … I sit down with the family and say, you know, I respect that you were trying to do what your family member asked you to do, but ultimately like it is our decision to decide what, what will actually help.” -Physician
Consult palliative care	“There really isn’t a great pathway if the surrogate disagrees with nephrology other than we would meet with them as the medical team and the nephrology team, and often we would bring in the palliative care team at that time as well.” -Physician “Physician team will do their best to, they’ll arrange care conferences with the family, involve palliative care, and do their best to explain their thought process of why the treatment might be futile or why it might be a good idea and they’ll lay all the decisions and all the facts out before the family or the surrogate decision maker, and, then that person is responsible for being able to make the decision based on what they know is what the patient would have wanted and what they believe is best for the patient.” -Nurse
Consult ethics	“We do invite our ethics team to be involved and take a look globally at the case, especially when we have two clinicians concerned that we may be offering something that causes more harm than benefit.” -Physician
Find a middle ground	“Between palliative care, ethics, and the ICU team, we can usually find some sort of negotiated way forward that is a midpoint between withdrawing or withholding or escalating care. And that I think is usually the goal rather, you know, to find some sort of middle ground that everybody can live with.” -Physician
Offer a second medical opinion	“We have, I mean if that doesn’t, if that’s not working, we’ve actually had, the physician stepped away from the patient care, and had one of their partners, come in and talk with the family and yeah, and if that second provider agrees then do the therapy or work with them and do a time limited therapy.” -Nurse
Go to court	“So this specific patient had been on ECMO for like 9 months and was not a transplant candidate and was awake and making his own decisions and wanted to stay alive for his daughter. And, his kidneys failed … but essentially we said that we would not be adding dialysis to his regimen because there was no exit strategy from ECMO, he couldn’t get a transplant, he’d been on for 9 months … the patient wanted to add dialysis anyway, because he did not want to die, he wanted to spend time with his daughter. … And ultimately, the family went to court and got a judge … [to say] that we had to restart it.” -Physician
Do not provide potentially inappropriate treatment	“We do not feel that it was is appropriate to offer this treatment. But we, will, of course continue to do all the things that we are already doing, but adding additional things is not within the medical realm of, appropriateness.” -Physician “However, we are not required to offer care that we view to be non-medically beneficial. We used to say that would be futile, but we don’t use that terminology anymore; the terminology has been changed to say non-medically beneficial care.” -Physician “We would tell the family, and then that would be kind of like we don’t give them any other alternatives to request or insist on things beyond that.” -Physician “We always are transparent with the family. And in this case yes, if that would be the only option, we would tell him that the patient does not meet criteria.” -Nurse
Provide potentially inappropriate treatment	“I think the default though truly amongst our culture, our hospital culture is to honor the surrogates which is even though we feel the clinical team feels that it’s an inappropriate treatment or therapy.” -Physician “I feel like normally the physicians do everything they can to honor the patient and family requests despite the medical recommendation.” -Nurse “I have seen patients still be offered either, like in this situation it would be CRRT or let's say ECMO, when a number of clinicians didn’t feel like it was gonna be life sustaining.” -Nurse
Approaching cases of an unrepresented patient without a surrogate decision-maker	
Treat as full code	“Typically, we continue assuming that the patient is full code, that we will do everything to keep the patient alive until we can identify—if not, someone directly involved with the patient.” -Nurse “Now, if they’re dying, and that person is circling the drain, then, then, and it, it becomes a moot point like it’s clearly futile, then, I would do what I would do for any patient and treat them until I can’t and that the assuming full code and then when I feel like we’ve reached the point of futility I’d stop.” -Physician “Let’s say we’ve done that and we cannot find and not a single decision maker. Then my understanding is the default’s gonna be that they, the patient undergoes a tracheostomy and peg, that we have to assume in this patient that we don’t know what their wishes are, that we have to be aggressive despite, you know, all of us believing that potentially this patient wouldn’t awaken.” -Physician
Seek information about patient	“But we do everything we can to find any information on the patient.” -Nurse
Search for decision-maker	“Contact anybody near the patient to see if there’s any—even neighbor who would be able to speak on the patient’s wishes.” -Nurse" So usually what they’ll actually do is try to get a private investigator and exhaust, like probably, you know, and we’ll probably engage in this scenario we usually engage ethics and legal to help, and they’ll usually make us do a pretty exhaustive [search].” -Physician “I believe in this scenario, what would happen is, what is called social work advantage. There will be a process that would establish, a spokesperson that would represent the interests of the patient, which would be, some, somebody from the community of, professional social workers that are not directly associated with the hospitals and that person would be deemed to be you know as a surrogate. This process of course takes some time but after that it’s completed, we would be really talking to this person as if this was a family member or if, if this was just a regular surrogate.” -Physician
Consult social work	“The first step we would get social work involved.” -Nurse “We have social work that would be closely involved in a case like this, if truly no one can be identified either as a surrogate decision maker.” -Physician “Require a couple of additional attempts from various social workers throughout the hospital to try and track down anybody.” -Physician
Consult hospital ethics and legal	“In this scenario we usually engage ethics and legal to help, and they’ll usually make us do a pretty exhaustive [search].” -Physician “This is one where we probably would get ethics involved typically. Okay. To help weigh in on the next step, obviously making every effort possible to find someone who can speak for this patient.” -Physician “I feel like generally we can find someone [to be a surrogate decision-maker]. But if we can’t, then we would be consulting ethics.” -Nurse “I heard that we no longer have an ethics committee. I’m not sure if that’s completely true. I think it’s kind of poorly communicated to nursing staff exactly what the ability of an ethics committee is, but in theory in the past I’ve seen us get ethics involved.” -Nurse
Involve police	“Get the police involved often the police come and fingerprint our patients at bedside and then try and determine an identity through that.” -Nurse “Sometimes we will also work with our police department if it’s an unidentified person.” -Physician “You go so far as trying to have the police fingerprint the patients, and so there’s a certain amount of, of work that goes into making sure that we do diligence to find somebody to speak for on behalf of, of this person.” -Physician
Seek state support	“But when you are talking about life sustaining treatment, like, eg, a trach and a peg and a long term SNF, that has to go to court and then the state will appoint a court appointed guardian, and then that person will make those decisions. And nearly always they will decide to trach and peg and go to a nursing facility.” -Physician “[If] it’s truly an issue after diligent searched by our social worker, then we would pursue guardianship for the patient. So it goes through the court process here, so it’s initiated by the social worker and then has to be approved, and then further decision-making goes through the actual proxy that’s appointed.” -Physician “Contacting the state to let them know that this is a person who is unidentified to us and the state would then assign a surrogate decision maker for the patient.” -Nurse
Two physician agreement	“There’s also two physicians that write in the chart that the, the diagnosis is terminal and the patient does not have, opportunity for meaningful recovery.” -Physician
Convene surrogate decision-making panel	“It would be the two of us as ICU attendings that examined and assessed the patient. We would then also have the patient’s bedside nurse, again, patient advocate, somebody from the ethics team, and the palliative care team. And we would then work to have a discussion about what we think the risks and benefits are. If that still reveals nobody, then we would have another decision to likely terminate care in this patient and withdraw life-sustaining support.” -Physician

ACLS = advanced cardiovascular life support; CRRT = continuous renal replacement therapy; DNI = do-not-intubate;DNR = do-not-resuscitate; ECMO = extracorporeal membrane oxygenation; SNF = skilled nursing facility.

No participant's response completely aligned with all requisite actions for approaching the 3 ethically challenging patient cases outlined in their hospital’s policies or state laws (e-Table 2). For example, policies for 2 hospital systems and state laws for all 3 hospital systems stated that clinicians must offer patients the opportunity to transfer to another institution if the patient or surrogate disagrees with a clinician decision to not provide or maintain LST. No participant at any hospital systems described that clinicians would offer transfer in these situations. In addition, no participant’s response completely aligned with all medical society consensus recommendations for approaching these scenarios.^12^^,^^13^ For example, in approaching cases of potentially inappropriate treatment, no clinician mentioned that clinicians would offer a review of the case by an interdisciplinary hospital committee or offer an appeals process to the patient or surrogate.

## Discussion

In this qualitative study of ICU nurses and physicians across 3 geographically diverse US hospital systems, we found that clinicians consistently lacked a detailed understanding about their hospital’s policies on withholding or withdrawing LST. Our qualitative design revealed important nuance in how hospital policies were considered useful (eg, prevent overtreatment of patients) and when policies were perceived as followed (eg, varied by specialty). Moreover, clinician perceptions about how patient care is approached in 3 ethically challenging patient cases aligned with neither the full processes outlined in their own hospital policies, state laws, nor medical society guidelines for how to address these scenarios.

To our knowledge, our data are the first to address a novel question: how does the specific content of hospital policies influence clinicians’ decisions to withhold or withdraw LST? Although prior investigations have identified how broad ethical principles within hospital policies align with do-not-resuscitate decisions, they have been limited in helping us understand whether clinician actions may be influenced by the specific detailed recommendations of their hospital policies.^10^ Our study adds to previous research by assessing how clinicians perceive hospitals’ policies are used in practice, whether clinicians perceive these policies address sociodemographic disparities in decision-making, and how clinician perceptions of approaching decisions to withhold or withdraw LST are aligned or misaligned with specific actions outlined in their hospital policies, state laws, and medical society consensus policy statements.^12^^,^^13^

Our findings suggest that hospital policies may have limited influence on clinician decisions to withhold or withdraw LST, a possibility that has important implications for patient care. First, limited hospital policy influence may contribute to the established variability in individual clinician approaches to decisions to withhold or withdraw LST, leading to differences in patient outcomes across clinicians.^1^^,^^4^, ^5^, ^6^ Our results suggest clinicians may approach decisions to withhold or withdraw LST differently across hospitals, the extent to which should be explored in future quantitative research. Second, lack of clinician awareness about policy guidance that prioritizes patient or surrogate voices may place patients at risk of not receiving care to which they are entitled. Hospital policies often provide expectations for actions that should occur in ethically challenging scenarios in which clinicians and patients or surrogates disagree with one another about withholding or withdrawing LST (eg, recommending that patients are offered a second clinical opinion or transfer to another institution).^7^ If clinicians are unaware of these expectations, patients may not receive these care options. Heterogeneity in preferences for LST identify that patients from vulnerable groups may be more likely to desire LST.^14^ This makes these groups disproportionately affected by clinician failures to offer actions outlined in their hospital policies, thus exacerbating existing sociodemographic disparities in decisions to withhold or withdraw LST.^15^ Sociodemographic disparities may be further worsened by variation in nurse and physician awareness about their existence because our study identified some nurses have not observed that these disparities exist.

Our findings also highlight an important medicolegal issue for clinicians facing decisions around withholding or withdrawing LST. Hospital policies may be viewed by courts as defining the standard of care that clinicians should provide to their patients, such that clinical practice inconsistent with local policy could be construed as a breach of professional duty to a patient.^16^^,^^17^ Especially in situations where clinician decisions to withhold or withdraw LST directly results in patient death, this possibility could place clinicians and hospital systems at risk for medical malpractice lawsuits.^18^

Because of the potential for patient and clinician harm if clinicians do not follow hospital policy recommendations, it is important to understand and address the underlying reasons for this misalignment. First, because our findings suggest that clinicians have limited knowledge about hospital policy content, efforts to educate clinicians about important policies might be helpful. Interactive educational strategies (eg, auditing clinician behavior and providing feedback) should be used rather than didactic lectures which have little to no effect on changing clinician behavior.^19^^,^^20^ More broadly, it is important to understand if efforts spent creating hospital polices, which may have little influence on clinician behavior, may be better allocated to other methods to promote consistency in patient care across clinicians, such as regularly recurring discussions among clinicians about recommended approaches to withhold or withdraw LST in ethically challenging scenarios. Hospital systems should urgently address whether clinician behavior aligns with hospital policy guidance within their institutions and address situations if misalignment exists. They should also explore whether limitations in clinician knowledge about hospital polices extend beyond policies addressing LST decisions.

### Strengths and Limitations

Our study has notable strengths, including the use of mixed inductive and deductive approaches to evaluate our data, which allowed us to leverage existing theories for how clinicians approach these situations using hospital policies and explore new insights that arose from the data outside of hospital policy guidance in ways that a survey cannot assess. Our study also has limitations. Because this qualitative study only assessed themes associated with clinician perceptions for how clinicians approach decisions to withhold or withdraw LST, future research is needed to quantitatively explore clinician behavior in practice, including how this behavior varies across professions and hospitals. Although we pursued diversity in participant recruitment, our results from clinicians at academic hospital systems may not reflect the experiences of clinicians at nonacademic hospital systems. Our work only assessed clinician perspectives, which may incompletely capture the variety of mechanisms in which hospital policies may influence clinician decision-making, such as through the assistance of legal or ethics consultants whose recommendations to clinicians about LST decisions may be influenced by hospital policies. Thus, future research should assess legal and ethics representatives’ perspectives. We did not explore reasons why some participants perceived that sociodemographic disparities did not exist in clinician decision-making, which should be pursued in future research.

## Interpretation

Our findings demonstrate that ICU nurses and physicians lacked detailed understanding about their hospitals’ policies that address withholding or withdrawing LST and identified important limitations with the applicability of these policies in practice. These findings suggest that hospital policies may have limited influence on clinician behavior in addressing decisions to withhold or withdraw LST in ethically challenging scenarios.

## Funding/Support

The authors have reported to *CHEST* that no funding was received for this study.

## Financial/Nonfinancial Disclosures

None declared.

## Supplementary Data

e-Online Data

Audio
