# Supplementary material for: The Influence of Hospital Policies on Clinicians’ Decisions to Withhold or Withdraw Life-Sustaining Treatment
Source: Chest. 2025 Jul 3;168(6):1446–58. doi: 10.1016/j.chest.2025.06.036 (PMC12833480; doi:10.1016/j.chest.2025.06.036)
Supplement: e-Online Data [file mmc1.docx]

**Online Supplement**

**e-Appendix 1**

**Semi-structured Interview Instrument** –

Begin by asking demographic questions from interview participants

1. Profession (physician [pulmonary-critical care; anesthesia critical care; surgical critical care; neuro critical care] or intensive care nurse (describe what unit- medical ICU, surgical ICU, etc)
2. Years in practice
3. Sex
4. Race
5. Hospital name (
6. Hospital location (city, state)
7. Hospital demographics (academic, community, religious, urban, rural, etc.)

*Today I am going to ask you about your experiences with seriously ill patients in the intensive care unit (ICU). Specifically, I want to talk with you about patients who are receiving or may receive life-sustaining treatments (LST) in this setting. By LST, I mean therapies for organ failures such as invasive mechanical ventilation, vasoactives, acute dialysis, extracorporeal membrane oxygenation (ECMO), and cardiopulmonary resuscitation (CPR).*

1. **WHO** are involved in decisions with withhold or withdraw LST at your hospital?

a. during ordinary patient care;

b. when disagreement among clinicians and patients/surrogates/families exist

Prompt for a and b if needed: patients?, surrogates? families?, physicians?, nurses?, ethics?, administration?, legal?, chaplaincy? racial/ethnic/socioeconomic diverse staff? community representative? a committee?)

*Now I am going to ask you questions that broadly address how decisions to withhold or withdraw life sustaining treatment are made at your hospital. Sometimes decisions to withhold or withdraw LST are associated with conflict between clinicians, patients and families. For the following questions, I want you to think about a set of hypothetical cases.*

*Case1: The first case involves a 50 year old patient with decompensated liver cirrhosis who is admitted to the ICU with hepatic encephalopathy requiring intubation and hypotension requiring vasoactive agents. The patient has been admitted to the hospital for 20 days and has not been alert during this period. They have previously undergone evaluation for liver transplantation and were deemed a noncandidate due ongoing alcohol use despite knowing alcohol use caused their cirrhosis. The patient has not completed previous advance directive paperwork. The patient* ***DOES*** *have a health care surrogate able and willing to make decisions on their behalf.*

*The patient’s renal function has significantly worsened secondary to hepatorenal syndrome. Their renal function does not improve with treatments for hepatorenal syndrome including diuresis, albumin and vasopressors.*

***While the patient can technically be placed on acute continuous renal replacement therapy (CRRT) for treatment of hepatorenal syndrome, the clinician judges this treatment to be “potentially inappropriate” because they view the harms of this treatment to outweigh the benefits for the patient. The clinician decides to not offer CRRT to the patient and informs the surrogate of this.***

***The patient’s surrogate disagrees with this decision and requests that the patient be placed on CRRT based on their understanding about how to treat renal failure in liver disease.*** *The surrogate explains the patient told them they wanted to extend their life in any way possible and the surrogate wants to honor this request.*

2. At your hospital, what is the typical hospital practice for approaching this patient scenario (i.e. “potentially inappropriate” treatment)?

*Case 2: The next hypothetical case is a 77-year-old patient with idiopathic pulmonary fibrosis who is admitted to the ICU with acute on chronic hypoxemic respiratory failure. The cause of their respiratory failure is believed to be solely due to irreversible damage from their pulmonary fibrosis. They are receiving maximum invasive mechanical ventilatory support and remain hypoxemic with an oxygen saturation of 70%. They have previously undergone evaluation for lung transplantation and were deemed a noncandidate due to age and ongoing inhaled tobacco use. The patient is neither awake nor alert and you do not believe that sedation can be safely lifted for them to communicate about their prognosis or care plans. The patient has not completed previous advance directive paperwork. The patient* ***DOES*** *have a health care surrogate able and willing to make decisions on their behalf.*

*Over the day the patient’s oxygen saturation continues to decrease despite maximum invasive mechanical ventilatory support. The clinician believes the patient will soon experience a cardiac arrest secondary to hypoxemia and that the cardiac arrest cannot be reversed with CPR.* ***Thus they view CPR to be physiologically futile for this patient and should not be performed.***

***The clinician informs the patient’s surrogate that the patient is going to die soon despite maximum medical intervention.*** ***The clinician explains to the surrogate that CPR will not work to bring the patient back to life and for this reason, they are going to place a DNR order.*** ***The patient’s surrogate disagrees with this recommendation and requests that the patient receive CPR.*** *The surrogate explains the patient told them they wanted to extend their life in any way possible and the surrogate wants to honor this request.*

3. At your hospital, what is the typical hospital practice for approaching this patient scenario (i.e., physiologic futility)?

*Case 4: The next hypothetical case involves a 30 year old patient who was admitted to the ICU after being found down on the street outside the hospital in cardiac arrest. The patient was without a pulse for at least 5 minutes before CPR was initiated, and CPR was continued for 45 minutes until the patient had sustained return of spontaneous circulation. The patient has now been in the ICU for one week following the cardiac arrest and remains intubated. Head imaging shows severe anoxic brain injury, but the patient does not meet criteria for death by neurological criteria per hospital policy criteria.* ***The patient DOES NOT wake up when sedation is lifted.*** ***After extensive evaluation, no one is able to determine the identity of this patient. This patient has no advance directive paperwork and no surrogate able and willing to speak on their behalf.***

4. At your hospital, what is the typical hospital practice for approaching this patient scenario (i.e. an unrepresented patient)?

*Now I am going to ask you questions about disparities in LST decision-making.*

5. What is your experience with racial/ethnic or other sociodemographic disparities in LST decision-making?

Probe: if interview respondent does not come up with disparities on their own: Recent studies have identified that clinicians disproportionately make unilateral decisions to withhold LST in certain vulnerable populations. For example, there is disproportionate use of unilateral do not resuscitate orders for Spanish speaking patients and disproportionate withholding of extracorporeal membrane oxygenation (ECMO) for patients who are female, insured by Medicaid, or live in low income neighborhoods.

6. How does your hospital approach racial/ethnic or other sociodemographic disparities in LST decision-making?

Follow up:

1. how are these disparities evaluated?
2. what barriers exist to evaluating these disparities (for example, is there difficulty collecting data in a registry to identify these disparities)?
3. what steps are taken to reduce these disparities?

7. At your hospital, how are clinician quality of life judgments used in decisions to withhold or withdraw LST? (for example, if a clinician determines a patient is unlikely to return to a “meaningful” life, they are not offered LST such as ECMO)

8. At your hospital, what factors influence clinical decisions about withholding or withdrawing LST?

**PLEASE DO NOT PROVIDE PROBES INITIALLY, LET INTERVIEW RESPONDENT ANSWER QUESTION 8 FIRST BEFORE PROVIDING PROBE EXAMPLES IF NEEDED.**

Probe: current hospital culture, your past training and experiences, hospital policy, international consensus guidelines, state law, frequent clinician turnover in ICU, minimizing conflict with patients and/or families, respecting patient values, etc.)

9. Returning to Case 1, which involved a patient with decompensated liver cirrhosis and the question of “potentially inappropriate” treatment, please answer the following: (*can reread Case 1 if needed)*

1. How do your hospital policies influence clinical practice in approaching this patient scenario, if at all?
2. What parts of your hospital policy are helpful in approaching this patient scenario?
3. What parts of your hospital policy are lacking in approaching this patient scenario?

10. Returning to Case 2, which involved a patient with idiopathic pulmonary fibrosis and the question of physiologic futility, please answer the following: (*can reread Case 2 if needed)*

1. How do your hospital policies influence clinical practice in approaching this patient scenario, if at all?
2. What parts of your hospital policy are helpful in approaching this patient scenario?
3. What parts of your hospital policy are lacking in approaching this patient scenario?

11. Returning to Case 3, which involved an unrepresented patient who experienced an out-of-hospital cardiac arrest, please answer the following: (*can reread Case 3 if needed)*

1. How do your hospital policies influence clinical practice in approaching this patient scenario, if at all?
2. What parts of your hospital policy are helpful in approaching this patient scenario?
3. What parts of your hospital policy are lacking in approaching this patient scenario?

12. How do your hospital policies influence clinical practice in approaching sociodemographic disparities in LST decision-making, if at all?

13. How do your hospital policies influence clinical practice in using clinician quality of life judgments in LST decision-making, if at all?

*Questions 14 is for intensivists only*

14. Can you give me an example of when your hospital policy influenced your clinical decisions about withholding and withdrawing LST?

Follow up: How do you believe your hospital policies support or impede you in approaching these decisions, if at all?

15. In your experience, how consistently do clinicians interpret and accurately apply your hospital’s policy when making decisions to withhold or withdraw LST?

16. How well do you know your hospital policies that address withholding and withdrawing LST?

Follow up: How did you learn these policies exist? Do you know owns this hospital policy?

Is there anything else you would like to share with me today?

**e-Table 1. Consolidated criteria for reporting qualitative studies (COREQ): 32-item checklist**

| **No. Item** | **Guide questions/description** | **Header and Paragraph** |
| --- | --- | --- |
| **Domain 1: Research team and reﬂexivity** |  |  |
| *Personal Characteristics* |  |  |
| 1. Interviewer/facilitator | Which author/s conducted the interview or focus group? | Data Collection paragraph 1 |
| 2. Credentials | What were the researcher’s credentials? E.g. PhD, MD | Authors |
| 3. Occupation | What was their occupation at the time of the study? | Data Collection paragraph 1 |
| 4. Gender | Was the researcher male or female? | Data Collection paragraph 1 |
| 5. Experience and training | What experience or training did the researcher have? | Data Collection paragraph 1 |
| *Relationship with participants* |  |  |
| 6. Relationship established | Was a relationship established prior to study commencement? | Data Collection paragraph 1 |
| 7. Participant knowledge of the interviewer | What did the participants know about the researcher? e.g. personal goals, reasons for doing the research | Supplement Semi-structure Interview Instrument paragraph two |
| 8. Interviewer characteristics | What characteristics were reported about the interviewer/facilitator? e.g. Bias, assumptions, reasons and interests in the research topic | Data Collection paragraph 1 |

| **Domain 2: study design** |  |  |
| --- | --- | --- |
| *Theoretical framework* |  |  |
| 9. Methodological orientation and Theory | What methodological orientation was stated to underpin the study? e.g. grounded theory, discourse analysis, ethnography, phenomenology, content analysis | Data Analysis paragraph 1 |
| *Participant selection* |  |  |
| 10. Sampling | How were participants selected? e.g. purposive, convenience, consecutive, snowball | Participants paragraph 1 |
| 11. Method of approach | How were participants approached? e.g. face-to-face, telephone, mail, email | Participants paragraph 1 |
| 12. Sample size | How many participants were in the study? | Results paragraph 1 |
| 13. Non-participation | How many people refused to participate or dropped out? Reasons? | Results paragraph 1 |
| *Setting* |  |  |
| 14. Setting of data collection | Where was the data collected? e.g. home, clinic, workplace | Data Collection paragraph 1 |
| 15. Presence of non-participants | Was anyone else present besides the participants and researchers? | n/a |
| 16. Description of sample | What are the important characteristics of the sample? e.g. demographic data, date | Results paragraph 1 |
| *Data collection* |  |  |
| 17. Interview guide | Were questions, prompts, guides provided by the authors? Was it pilot tested? | Supplement Semi-structure Interview Instrument and Semi-structured Interviews paragraph two |
| 18. Repeat interviews | Were repeat interviews carried out? If yes, how many? | **n/a** |
| 19. Audio/visual recording | Did the research use audio or visual recording to collect the data? | Data Collection paragraph one |
| 20. Field notes | Were ﬁeld notes made during and/or after the inter view or focus group? | n/a |
| 21. Duration | What was the duration of the interviews or focus group? | Results paragraph 1 |
| 22. Data saturation | Was data saturation discussed? | Data analysis paragraph 1 |
| 23. Transcripts returned | Were transcripts returned to participants for comment and/or correction? | n/a |
| **Domain 3: analysis and ﬁndings** |  |  |
| *Data analysis* |  |  |
| 24. Number of data coders | How many data coders coded the data? | Data Analysis paragraph 1 |
| 25. Description of the coding tree | Did authors provide a description of the coding tree? | Data Analysis paragraph 1 |
| 26. Derivation of themes | Were themes identiﬁed in advance or derived from the data? | Data Analysis paragraph 1 |
| 27. Software | What software, if applicable, was used to manage the data? | Data Analysis paragraph 1 |
| 28. Participant checking | Did participants provide feedback on the ﬁndings? | n/a |
| *Reporting* |  |  |
| 29. Quotations presented | Were participant quotations presented to illustrate the themes/ﬁndings? Was each quotation identiﬁed? e.g. participant number | Yes, throughout Results section |
| 30. Data and ﬁndings consistent | Was there consistency between the data presented and the ﬁndings? | Yes, throughout Results section |
| 31. Clarity of major themes | Were major themes clearly presented in the ﬁndings? | Yes, throughout Results section |
| 32. Clarity of minor themes | Is there a description of diverse cases or discussion of minor themes? | Yes, throughout Results section |

**e-Table 2.** **Select Required Core Hospital Policy Components in Addressing Clinician Decisions to Withhold or Withdraw Life-sustaining Treatment Without Patient/Surrogate Agreement**

| **Core component** | **Hospital system A** | **Hospital system B** | **Hospital system C** |
| --- | --- | --- | --- |
| **Inform patient/surrogate of the decision to withhold or withdraw life-sustaining treatment** | Yes | Yes | Yes |
| **Offer option of receiving other types of care (e.g., palliative care, hospice care)** | Yes | No | Yes |
| **Offer transfer to another institution** | Yes | Yes | No |
